# Association of Left Atrium Remodeling With Major Adverse Cardiovascular Events in Asymptomatic Type 2 Diabetes Patients With Early Chronic Kidney Disease

**DOI:** 10.31083/RCM27247

**Published:** 2025-05-21

**Authors:** Mingxia Gong, Min Xu, Suoya Pan, Shu Jiang, Xiaohong Jiang

**Affiliations:** ^1^Department of Echocardiography, The Third Affiliated Hospital of Soochow University, 213003 Changzhou, Jiangsu, China; ^2^Department of Cardiology, The Third Affiliated Hospital of Soochow University, 213003 Changzhou, Jiangsu, China; ^3^Department of Endocrinology, The Third Affiliated Hospital of Soochow University, 213003 Changzhou, Jiangsu, China

**Keywords:** diabetic nephropathy, left atrial volume, left atrial strain, four-dimensional automatic left atrial quantification (4D Auto LAQ), major adverse cardiovascular events

## Abstract

**Background:**

This study aimed to use four-dimensional automatic left atrial quantification (4D Auto LAQ) to quantitatively evaluate the morphological and functional changes in the left atrium (LA) in asymptomatic type 2 diabetes mellitus (T2DM) patients with early chronic kidney disease (CKD), and explore its correlation with major adverse cardiovascular event (MACE) occurrence.

**Methods:**

This study enrolled patients with asymptomatic T2DM complicated with early CKD. Then, 4D-Auto LAQ was used to evaluate LA volume index (minimum, maximum, pre-ejection) and LA longitudinal and circumferential strains during each of the three LA phases: reservoir, conduit, and contraction. The primary endpoint for follow-up was defined as the first occurrence of nonfatal acute myocardial infarction, stroke, congestive heart failure, or cardiac death. Univariate and multivariate Cox proportional hazard analyses were used to evaluate the correlation between LA parameters and the MACEs in T2DM patients with early CKD.

**Results:**

A total of 361 patients were analyzed (mean age, 59.51 ± 11.17 years). During a median follow-up period of 47 months (interquartile range, 17–59 months), MACEs occurred in 70 patients. After adjusting for various clinical and echocardiographic predictors, increased LA volume and impaired reservoir function (ResF) were each independently associated with the primary endpoint: Left atrium minimum volume index (LAVImin) had an adjusted hazard ratio (HR) of 1.21 (95% confidence interval (CI), 1.08–1.35; *p* = 0.010), whereas left atrium longitudinal strain during the reservoir phase (LASr) had an adjusted HR of 0.81 (95% CI, 0.74–0.89; *p* < 0.001). Univariate and multivariate Cox regression analyses indicated that the cumulative incidence of MACEs was significantly greater in patients with LAVImin >16.9 mL/m^2^ than in those with LAVImin ≤16.9 mL/m^2^ (HR, 2.25; 95% CI, 1.03–6.39; *p* = 0.005). Furthermore, patients with a LASr <18.5% faced a markedly elevated risk of MACEs—nearly fourfold greater than individuals with a LASr ≥18.5% (HR, 3.95; 95% CI, 1.76–8.86; *p* < 0.001).

**Conclusions:**

An enlarged left atrium (LAVImin) and impaired ResF (LASr) are strongly associated with long-term outcomes in T2DM patients complicated with early CKD. LASr showed the strongest associations with the occurrence of MACEs.

## 1. Introduction

Diabetic nephropathy (DN) is an important and serious microvascular complication of diabetes mellitus (DM), and its incidence has been increasing over the years [1]. Research conducted in European and American countries indicates that 20% to 40% of individuals with diabetes may progress to DN [2, 3]. Whereas the prevalence of DN in Chinese patients with type 2 diabetes mellitus (T2DM) is 21.8% [4, 5], the 2023 guidelines proposes that renal involvement is the main criterion for the evaluation of serious target organ impairment in diabetes patients [6]. Compared with individuals with uncomplicated diabetes, those with DN exhibit a significantly elevated risk of cardiovascular events and mortality [7]. Even in the early stages, the incidence of cardiovascular disease (CVD) is considerable [4]. A study revealed that patients with diabetes and kidney disease had a standardized mortality rate of 31.1% [8] and in the absence of nephropathy, mortality in diabetic patients does not differ greatly from that in the general population. However, once kidney disease is present, excess cardiovascular mortality is observed. Patients with DN frequently present with comorbidities, including being overweight or obese; suboptimally managed hyperglycemia; hypertension; dyslipidemia; a hypercoagulable state; and an elevated risk of hypoglycemia. These conditions collectively contribute to an increased susceptibility to CVD. The onset of DN is insidious, often eluding early detection. As the urine albumin-to-creatinine ratio (UACR) increases, or the estimated glomerular filtration rate (GFR) decreases, there is a significant increase in the risk of CVD and related mortality among individuals with T2DM [9]. Elevated urinary albumin excretion is correlated with adverse renal and cardiovascular outcomes [8, 10]. In the early stages of DN, when only pathological changes such as increased GFR and microalbuminuria are promptly addressed with appropriate treatment, the patient’s condition may still be reversible [11]. Therefore, the early identification and management of alterations in the mechanical function of the DN heart are paramount. This finding holds critical importance in preventing or delaying the onset of chronic renal damage and CVD associated with diabetes.

Recent research on DN-related CVD has focused predominantly on left ventricular (LV) function. Jørgensen *et al*. [12] reported that T2DM patients with microalbuminuria (MA) experience left ventricular diastolic dysfunction (LVDD). However, research on left atrium (LA) correlations in patients with DN is limited. LA dysfunction in patients with chronic kidney disease (CKD) is independently regulated by renal function, and LA alterations may consequently increase the activation of the renin‒angiotensin‒aldosterone pathway, causing myocardial fibrosis in CKD and the development of atrial myopathy [13]. Microvascular damage in patients with DN leads to inadequate myocardial perfusion, decreased atrial wall elasticity, diminished cardiac filling function, and a compensatory increased workload in the LA to maintain normal LV filling. LA remodeling typically occurs at an earlier stage [14]. LA strain and left atrial volume index (LAVI) are more sensitive parameters than traditional echocardiographic parameters and left ventricular strain in patients with early CKD. LA strain and LAVI may be useful for detecting myocardial involvement in patients with stage 3 CKD [13].

Compared with those of the LV, the thin wall, complex geometry, short muscle fibers, and irregular arrangement of the left atrium pose challenges to two-dimensional measurements of volume and strain, limiting accuracy and repeatability [15]. Previous studies have investigated left atrial function in patients with renal insufficiency via various methods, such as real-time three-dimensional echocardiography (RT-3DE), left atrial volume tracking (LAVT), two-dimensional speckle-tracking imaging (2D-STI), three-dimensional speckle-tracking imaging (3D-STI) and velocity vector imaging (VVI) [16, 17]. However, each method has its limitations. In contrast, four-dimensional automatic left atrial quantification (4D Auto LAQ), a new tool for analyzing LA structure and function via 3DE, has distinct advantages in the ‘one-stop-shop’ comprehensive evaluation of LA volume, emptying fraction, and strain [18]. 4D Auto LAQ has high sensitivity, repeatability, and accuracy [19], demonstrating angle- independence. It is more sensitive to LA functional impairment and can provide more valuable information for clinical diagnosis [18, 20].

The objective of this study was to investigate the relationship between the morphological and functional changes in the LA and the outcome of major adverse cardiovascular events (MACEs) in asymptomatic T2DM patients with early CKD.

## 2. Methods

### 2.1 Study Population

**Fig. 1.**
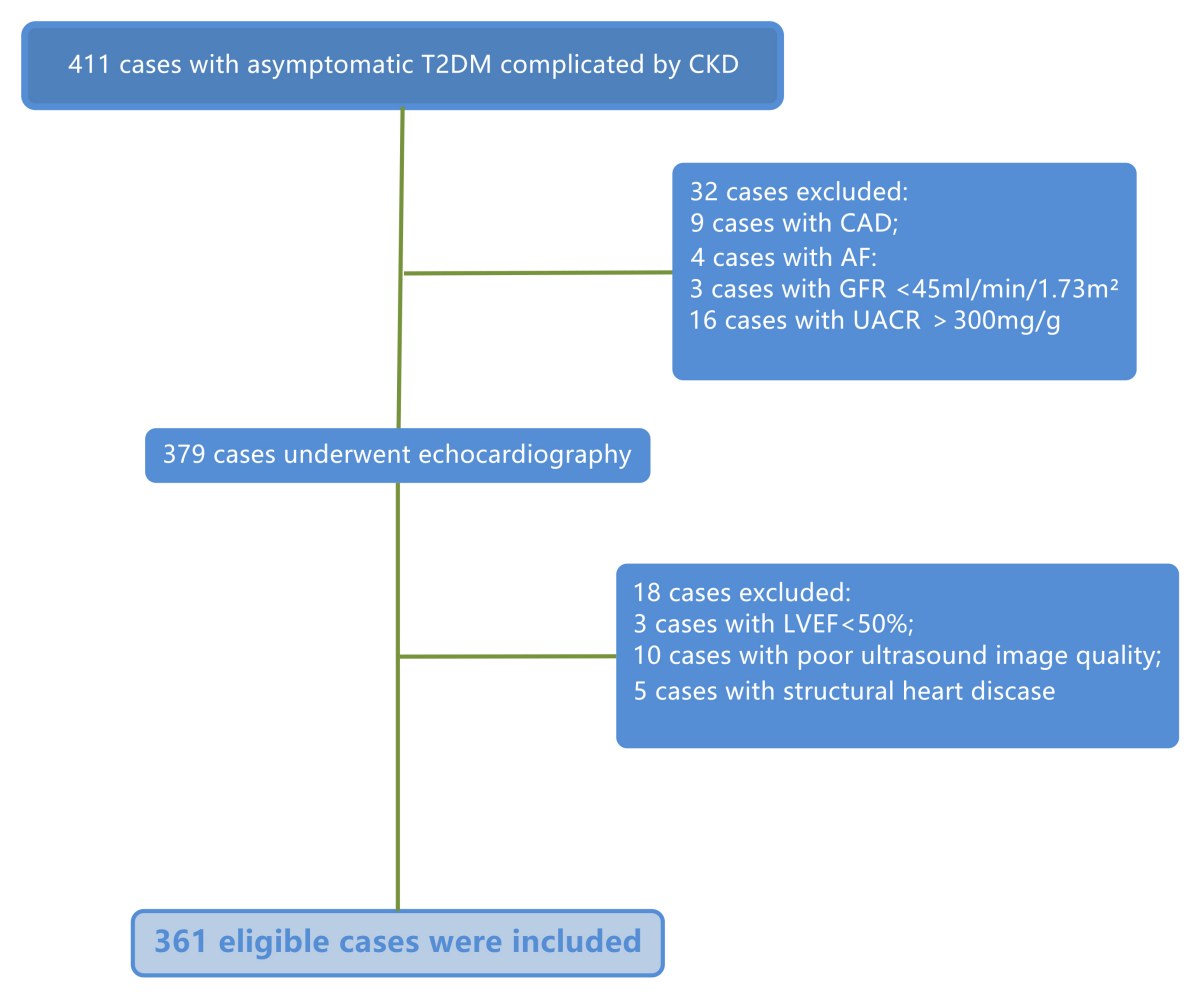
**Flow diagram**. T2DM, type 2 diabetes mellitus; CKD, chronic kidney disease; CAD, coronary artery disease; AF, atrial fibrillation; GFR, glomerular filtration rate; UACR, urine albumin-to-creatinine ratio; LVEF, left ventricular ejection fraction.

This study selected patients with DN with asymptomatic T2DM combined with microalbuminuria and who were hospitalized between January 2018 and January 2020. The inclusion criteria for patients were as follows: (1) were ≥18 years old; (2) met all the diagnostic criteria for T2DM [21] and DN [22]; (3) UACR ranging from 30 to 300 mg/g and a GFR ≥45 mL/min/1.73 m^2^; (4) coronary stenosis <50% as confirmed by coronary computed tomography or coronary angiography. The exclusion criteria were as follows: (1) had a history of CVD, including congenital heart disease, cardiomyopathy, coronary artery disease, heart failure (HF), atrial fibrillation (AF), or valvular heart disease; (2) previous macrovascular complications of diabetes, such as peripheral vascular disease and stroke; (3) pregnancy; (4) other significant comorbidities, such as malignant tumors, thyroid dysfunction, liver or kidney insufficiency, rheumatic immune disease or major mental illness; and (5) had poor-quality ultrasound images, left ventricular ejection fraction (LVEF) <50%, or New York Heart Association (NYHA) functional class II or higher (Fig. 1). This study was approved by the hospital ethics committee, and all patients provided informed consent.

#### 2.1.1 Candidate Variables

The clinical data of the patients, including age, sex, body mass index (BMI), and family history of diabetes, were systematically collected. Biochemical measurements, including fasting blood glucose (FBG), glycated hemoglobin A1c (HbA1c), total cholesterol (TC), triacylglycerol (TG), high-density lipoprotein cholesterol (HDL-C), low-density lipoprotein cholesterol (LDL-C), serum creatinine (CR), GFR, UACR, and B-type natriuretic peptide (BNP), were measured under fasting conditions in the morning. After a 20-minute rest period, systolic blood pressure (SBP) and diastolic blood pressure (DBP) were measured three times, and the average values were recorded. A comprehensive medical history was also obtained from each patient.

#### 2.1.2 Diagnosis of DN

Diagnostic criteria: The diagnosis of DN adhered to the guidelines established by the 2020 Global Kidney Disease Prognosis Organization [22]. Patients with a UACR exceeding 30 mg/L and/or a GFR below 60 mL/min/1.73 m^2^ persisting for more than 3 months were included, provided they did not have nondiabetic CKD, such as membranous nephropathy, immunoglobulin A (IgA) nephropathy, focal segmental glomerulosclerosis, confirmed by renal biopsy, or diabetic ketoacidosis.

### 2.2 Echocardiographic Image Acquisition and Analysis

#### 2.2.1 Echocardiographic Image Acquisition

All patients underwent transthoracic echocardiography (TTE) via the GE Vivid E95 system with M5S (frequency: 2.0–4.5 MHz) and 4 V probes (frequency: 1.5–4 MHz) (GE Vingmed Ultrasound As, Horten, Norway). Patients were positioned in the left lateral decubitus, connected to a 12-lead electrocardiogram (ECG). In accordance with the 2015 American Society of Echocardiography (ASE) guideline [23], we acquired the following parameters: Left ventricular end-diastolic and end-systolic diameters (LVEDd, LVESd), the LVEF calculated via biplane Simpson’s method, and diastolic function (mitral E and A velocities, the E/A ratio, the medial E/e’ ratio, etc.). LV global longitudinal strain (LVGLS) was obtained via speckle-tracking echocardiography. With three-plane imaging mode, a 4 V probe was used to capture images of six consecutive cardiac cycles at an image frame rate exceeding 40 frames per second. Three-dimensional images of the adjacent 6 cardiac cycles were obtained, and the image frame rate was >40 frames/s.

All patient images were digitally stored for subsequent analysis. Image acquisition and analysis were completed independently by two or more attending physicians, and the average values were taken.

#### 2.2.2 Image Analysis

Image analysis was performed via the use of the 4D Auto LAQ software within Echo PAC (203, 204) (GE Vingmed Ultrasound As, Horten, Norway), which automatically tracks the endocardium and reconstructs four-dimensional dynamic images of the LA. Various parameters including the LA maximum volume index (LAVImax), pre-ejection volume index (LAVIpre), minimum volume index (LAVImin), LA longitudinal strain during the reservoir phase (LASr) and circumferential strain during the reservoir phase (LASr-c), LA longitudinal strain during the conduit phase (LAScd) and circumferential strain during the conduit phase (LAScd-c), LA longitudinal strain during the contraction phase (LASct) and circumferential strain during the contraction phase (LASct-c), as well as the left atrial ejection fraction (LAEF), were measured.

### 2.3 Follow-up

Follow-up periods for patients diagnosed with the early DN ranged from 3 to 80 months, with a median follow-up duration of 47 months. Follow-ups were conducted every 6 to 12 months via telephone or outpatient examination, if necessary, patients were asked to return to the hospital for reexamination. The primary endpoint event was MACEs, defined as a composite outcome of cardiogenic death, hospitalization related to HF, nonfatal acute myocardial infarction, and stroke. Patients were categorized based on the occurrence of MACEs.

### 2.4 Statistical Analysis

Statistical analysis was performed via SPSS 22.0 software (IBM Corp., Armonk, NY, USA) and R software (http://www.R-project.org, version 3.4.3). The Shapiro-Wilk test was used to assess data normality. Normally distributed continuous variables are expressed as the mean ± standard deviation ($\bar{x}$ ± s), whereas skewed distributions are reported as the median (interquartile range, IQR: Q1, Q3). Categorical variables are presented as percentages, and group comparisons were performed via chi-square tests or Fisher’s exact probability methods. Time-to-event analysis was conducted via Harrell’s method to calculate areas under the curves for associations between variables and outcomes. Univariate and multivariate Cox proportional hazard models, along with log-rank statistic, were employed to evaluate the relationships between LA parameters and the MACEs. Three regression models were analyzed: Model 1: unadjusted for covariates; Model 2: preliminarily adjusted model, adjusted for age, SBP, smoking, HbA1c, triglycerides, CR and GFR; Model 3: fully adjusted model, adjusted for LVEDd and global longitudinal strain (GLS) in addition to Model 2 hazard ratios (HRs) and corresponding 95% confidence intervals (95% CIs) were calculated for each model. A *p*-value < 0.05 was considered statistically significant. Cumulative event rates were assessed via Kaplan-Meier analysis, with log-rank *p*-values reported. Subgroup analysis and interaction effects analysis were conducted to further evaluate the associations between MACEs and the LA parameters across different subgroups. Subgroups were stratified by age, sex, duration of diabetes, and filtration rate.

## 3. Results

### 3.1 Study Population

#### 3.1.1 Comparison of Basic Characteristics

In this study, a total of 361 asymptomatic patients with early DN were enrolled, comprising 213 men (59.0%) with an average age of 59.51 ± 11.17 years. The average duration of diabetes was 9.31 ± 4.97 years. During a median follow-up period of 47 months (IQR, 17–59 months), 70 patients experienced MACEs: 36 cardiac hospitalizations (29 with acute coronary syndrome, 7 with HF), 28 ischemic strokes, 6 with hemorrhagic strokes, and 0 with cardiac death. The patients’ baseline characteristics are presented in Table 1. There were significant differences in age, SBP, smoking history, triglyceride levels, CR rates and estimated glomerular filtration rates (eGFRs) between the two groups of patients with DN with and without MACEs (all *p* < 0.05).

**Table 1.** **Characteristics of clinical data, biochemistry and medication of the study population**.

Clinical characteristics	All patients (N = 361)	Patients without MACE (n = 291)	Patients with MACE at follow-up (n = 70)	*p*-value
Age (years)	59.51 ± 11.17	58.93 ± 10.51	61.91 ± 9.13	**0.028**
Men, n (%)	213 (59.00)	173 (59.45)	40 (57.14)	0.540
BMI (kg/m^2^)	24.86 ± 3.45	24.77 ± 3.15	25.36 ± 3.53	0.156
SBP (mmHg)	140.23 ± 16.92	138.31 ± 11.51	147.36 ± 15.67	**0.005**
DBP (mmHg)	86.17 ± 9.04	85.63 ± 8.92	88.02 ± 9.33	0.071
Current smokers n (%)	86 (23.82)	64 (21.99)	22 (31.42)	**0.007**
Family history of diabetes, n (%)	73 (20.22)	59 (20.27)	14 (20.00)	0.725
Duration of diabetes (years)	9.31 ± 4.97	9.19 ± 3.89	9.94 ± 6.91	0.238
Blood examination				
FBG (mmol/L)	9.52 ± 2.35	9.56 ± 1.95	9.42 ± 2.92	0.084
PBG (mmol/L)	13.71 ± 3.32	13.78 ± 3.11	13.66 ± 3.46	0.592
HbA1c (%)	9.45 ± 2.95	9.67 ± 2.19	9.32 ± 3.81	0.257
Total cholesterol (mmol/L)	4.92 ± 1.35	4.81 ± 1.13	5.02 ± 1.94	0.542
Triglyceride (mmol/L)	2.37 (1.21–3.59)	2.21 (1.15–2.94)	2.99 (1.25–3.74)	**0.050**
HDL (mmol/L)	1.05 ± 0.32	1.04 ± 0.25	1.08 ± 0.39	0.402
LDL (mmol/L)	2.84 ± 0.92	2.81 ± 0.89	3.03 ± 0.98	0.541
BNP (pg/dL)	73.96 ± 5.32	72.18 ± 3.62	82.41 ± 7.18	0.257
CR (µmol/L)	98.01 ± 11.31	93.03 ± 10.26	103.16 ± 11.9	< **0.001**
UACR (mg/g)	102.48 (13.34–201.67)	99.71 (10.51–187.53)	107.11 (52.30–239.45)	0.413
eGFR (mL/min/1.73 m^2^)	81.44 ± 19.39	84.28 ± 14.84	67.56 ± 20.78	< **0.001**
Medications				
Insulin, n (%)	93 (25.76)	74 (25.42)	19 (27.14)	0.249
Metformin n (%)	223 (61.77)	177 (60.82)	46 (65.71)	0.355
Sulfonylureas, n (%)	150 (41.55)	119 (40.89)	31 (44.28)	0.418
Glitazones, n (%)	80 (22.16)	64 (21.99)	16 (22.85)	0.251
ACE/ARB, n (%)	181 (50.13)	141 (48.45)	40 (57.14)	0.095
Calcium channel blocker, n (%)	65 (18.01)	49 (16.83)	16 (22.85)	0.503
Diuretics, n (%)	99 (27.42)	79 (27.14)	20 (28.75)	0.524
Beta-blocker , n (%)	71 (19.66)	57 (19.58)	14 (21.42)	0.359
Statins, n (%)	144 (39.88)	116 (39.86)	28 (40.00)	0.435

Abbreviations: BMI, body mass index; SBP, systolic blood pressure; DBP, diastolic blood pressure; FBG, fasting blood glucose; PBG, postprandial blood glucose; HbA1c, glycated hemoglobin A1c; HDL, high-density lipoprotein; LDL, low-density lipoprotein; BNP, B-type natriuretic peptide; CR, serum creatinine; UACR, urine albumin-to-creatinine ratio; eGFR, estimated glomerular filtration rate; ACE, angiotensin converting enzyme; ARB, aldosterone receptor blocker; MACE, major adverse cardiovascular event. The data highlighted in boldface were found to be statistically significant.

#### 3.1.2 Comparison of Echocardiographic Parameters in Patients With DN

Compared with those in patients without MACEs, there were significant differences in LA volume (LAVImin, LAVImax) and strain (LASr, LAScd, LASr-c, and LAScd-c) in patients with MACEs (*p* < 0.05), whereas there were no statistically significant differences in LVESd, interventricular septal thickness (IVS), left ventricular posterior wall thickness (LVPW), LVEF, GLS, E/A, or E/e’ (Table 2).

**Table 2.** **Echocardiographic characteristics stratified by presence or absence of cardiovascular adverse events**.

Echocardiographic parameter	All patients (N = 361)	Patients without MACE (n = 291)	Patients with MACE at follow-up (n = 70)	*p*-value
LVEDd (mm)	52.25 ± 13.82	51.17 ± 13.82	54.32 ± 13.37	**0.023**
LVESd (mm)	21.79 ± 5.76	20.89 ± 5.47	22.23 ± 6.05	0.113
IVS (mm)	11.28 ± 2.56	11.14 ± 2.76	11.43 ± 2.08	0.445
LVPW (mm)	11.13 ± 1.89	10.98 ± 1.79	11.21 ± 1.95	0.307
LVEF (%)	58.97 ± 5.12	59.13 ± 4.83	58.67 ± 5.32	0.647
GLS (%)	–16.92 ± 2.66	–17.21 ± 2.59	–15.93 ± 2.72	0.067
E/A	0.81 ± 0.21	0.80 ± 0.19	0.83 ± 0.16	0.987
E/e’	10.17 ± 2.92	10.66 ± 2.03	9.97 ± 2.77	0.203
LAVImin (mL/m^2^)	17.14 ± 3.83	16.86 ± 3.79	18.91 ± 3.83	< **0.001**
LAVImax (mL/m^2^)	32.37 ± 6.92	31.99 ± 7.11	33.52 ± 5.97	**0.023**
LAVIpre (mL/m^2^)	25.45 ± 5.38	25.29 ± 5.04	25.85 ± 5.92	0.867
LAEF (%)	51.98 ± 6.21	52.18 ± 5.71	50.97 ± 6.84	0.361
LASr (%)	20.25 ± 4.56	21.02 ± 4.11	16.19 ± 4.96	< **0.001**
LAScd (%)	–9.48 ± 3.85	–9.86 ± 3.77	–7.46 ± 3.98	< **0.001**
LASct (%)	–11.81 ± 7.33	–11.11 ± 3.29	–9.41 ± 3.68	< **0.001**
LASr-c (%)	29.34 ± 6.96	30.07 ± 7.53	25.7 ± 6.85	< **0.001**
LAScd-c (%)	–11.64 ± 4.15	–11.98 ± 4.84	–9.87 ± 3.29	**0.002**
LASct-c (%)	–17.32 ± 6.55	–17.58 ± 7.36	–15.78 ± 6.09	**0.004**

Abbreviations: LVEDd, left ventricular end-diastolic diameter; LVESd, left ventricular end-systolic diameter; IVS, interventricular septal thickness; LVPW, left ventricular posterior wall thickness; LVEF, left ventricular ejection fraction; GLS, global longitudinal strain; E/A, ratio velocity E and velocity A; E/e’, ratio of transmitral Doppler early peak velocity (E) and tissue Doppler average early diastolic mitral annulus velocity (e’); LAVImin, left atrium (LA) minimum volume index; LAVImax, LA maximum volume index; LAVIpre, LA pre-ejection volume index; LAEF, LA ejection fraction; LASr, LA longitudinal strain during the reservoir phase; LAScd, LA longitudinal strain during the conduit phase; LASct, LA longitudinal strain during the contraction phase; LASr-c, LA circumferential strain during the reservoir phase; LAScd-c, LA circumferential strain during the conduit phase; LASct-c, LA circumferential strain during the contraction phase. The data highlighted in boldface were found to be statistically significant.

### 3.2 Associations with the Major Adverse Cardiovascular Events

The optimal cutoffs for LA parameters in relation to the MACE were 16.9 mL/m^2^ for LAVImin, 32.2 mL/m^2^ for LAVImax, 18.5% for LASr, and 27% for LASr-c (areas under the curve, 0.633, 0.513, 0.774, and 0.713, respectively) (see **Supplementary Table 1**). The clinical features and echocardiographic indices of patients stratified by cutoffs of LA parameters are presented in Table 3. In the univariate analysis, age, SBP, smoking status, HbA1c levels, triglycerides, CR, GFR, LVEDd, LVGLS, LA volumes, and LA strain were all significantly associated with the primary endpoint; conversely, sex, BMI, DBP, family history of diabetes, LVESd, LVEF, GLS, E/A, and E/e’ showed no such associations (refer to Table 4 for LA parameters; see **Supplementary Table 2** for clinical and other echocardiographic metrics).

**Table 3.** **Clinical and echocardiographic characteristics of patients stratified by LA parameters (total N = 361)**.

	Total (n = 361)	LAVImin		LAVImax		LASr		LASr-c	
		≤16.9 mL/m^2^ (n = 194)	>16.9 mL/m^2^ (n = 167)	≤32.2 mL/m^2^ (n = 186)	>32.2 mL/m^2^ (n = 175)	≥18.5% (n = 187)	<18.5% (n = 174)	≥27% (n = 185)	<27% (n = 176)
Age (years)	59.51 ± 11.17	58.45 ± 11.35	60.75 ± 10.43*	56.93 ± 11.94	59.39 ± 10.31	58.03 ± 10.96	60.99 ± 10.86	58.70 ± 11.08	60.36 ± 10.84
Men, n (%)	213 (59.00)	106 (54.64)	107 (64.07)	116 (62.36)	97 (55.4)	104 (55.61)	109 (62.64)	105 (56.76)	108 (61.36)
BMI (kg/m^2^)	24.86 ± 3.45	25.52 ± 3.14	24.12 ± 3.33	26.04 ± 3.35	23.78 ± 3.02	25.09 ± 3.11	24.73 ± 3.519	24.88 ± 2.98	24.84 ± 3.62
SBP (mmHg)	140.23 ± 16.92	138.54 ± 14.87	142.45 ± 18.85*	138.98 ± 16.01	141.63 ± 17.82*	140.12 ± 15.73	140.77 ± 18.41	140.68 ± 16.34	140.01 ± 17.55
Current smokers n (%)	87 (24.09)	49 (25.25)	38 (22.75)	48 (25.80)	39 (22.28)	41 (21.92)	46 (26.44)	42 (22.70)	45 (25.57)
Duration of diabetes (years)	9.31 ± 4.97	8.89 ± 6.93	9.93 ± 7.21	8.97 ± 6.93	9.67 ± 6.91	8.823 ± 6.542	8.828 ± 6.54	9.27 ± 6.71	9.47 ± 7.45
HbA1c (%)	9.45 ± 2.95	9.49 ± 2.20	9.40 ± 2.80	9.64 ± 2.26	9.47 ± 2.74	9.28 ± 2.29	9.44 ± 2.64	9.41 ± 2.34	9.489 ± 2.65
Triglyceride (mmol/L)	2.37 (1.21–3.59)	2.39 (1.18–3.75)	2.25 (1.12–3.37)	2.3 (1.15–3.67)	2.27 (1.14–3.25)	2.32 (1.17–3.62)	2.39 (1.21–3.79)	2.19 (1.11–3.87)	2.48 (1.19–3.52)
Serum creatinine (µmol/L)	98.01 ± 11.31	85.57 ± 27.85	107.51 ± 30.86^#^	86.34 ± 30.76	105.67 ± 32.75^#^	86.21 ± 30.37	104.17 ± 29.66^#^	83.6 ± 26.56	108.34 ± 30.79^#^
eGFR (mL/min/1.73 m^2^)	81.44 ± 19.39	95.24 ± 28.02	65.59 ± 16.72^#^	95.29 ± 34.63	65.46 ± 20.51^#^	94.14 ± 30.39	69.61 ± 18.38^#^	95.40 ± 29.01	66.87 ± 16.60^#^
LVEDd (mm)	52.25 ± 13.82	48.34 ± 13.39	56.52 ± 13.36*	49.93 ± 14.35	56.13 ± 13.51*	51.13 ± 13.4	52.98 ± 14.50	49.92 ± 13.75	54.22 ± 13.89*
GLS (%)	–16.92 ± 2.66	–17.15 ± 2.72	–16.76 ± 2.65	–17.28 ± 2.62	–16.55 ± 2.79	–17.34 ± 2.80	–16.60 ± 2.61	–17.26 ± 2.76	–16.67 ± 2.59
LAVImin (mL/m^2^)	17.14 ± 3.83	14.50 ± 1.34	20.1 ± 3.02^#^	14.97 ± 2.09	20.10 ± 3.46^#^	15.64 ± 2.85	18.5 ± 3.72^#^	15.39 ± 2.65	18.99 ± 3.55^#^
LAVImax (mL/m^2^)	32.37 ± 6.92	28.41 ± 4.21	36.97 ± 5.79^#^	27.45 ± 3.23	38.12 ± 5.39^#^	30.03 ± 5.81	34.72 ± 6.592^#^	18.99 ± 3.56	35.24 ± 6.504^#^
LAEF (%)	51.98 ± 6.21	53.96 ± 5.68	50.19 ± 5.75	51.59 ± 6.19	52.39 ± 6.08	53.25 ± 6.17	51.34 ± 5.66	53.38 ± 5.47	50.994 ± 6.30
LASr (%)	20.25 ± 4.56	20.96 ± 4.01	18.06 ± 3.65^#^	21.38 ± 4.89	18.11 ± 3.99^#^	22.6 ± 3.21	16.40 ± 1.98^#^	21.66 ± 3.92	17.36 ± 3.04^#^
LAScd (%)	–9.48 ± 3.85	–10.04 ± 3.17	–8.09 ± 3.34^#^	–10.13 ± 3.37	–8.82 ± 3.61^#^	–10.82 ± 3.11	–7.33 ± 2.63^#^	–10.49 ± 3.16	–7.16 ± 3.02^#^
LASct (%)	–11.81 ± 7.33	–11.14 ± 3.54	–10.16 ± 3.17	–11.22 ± 3.85	–10.24 ± 2.89	–11.76 ± 3.46	–9.69 ± 3.08	–11.32 ± 3.57	–10.02 ± 3.10
LASr-c (%)	29.33 ± 6.96	31.44 ± 4.68	26.91 ± 4.31^#^	31.69 ± 5.61	26.94 ± 4.44^#^	31.57 ± 4.84	26.93 ± 4.013^#^	32.68 ± 4.24	24.81 ± 2.35^#^
LAScd-c (%)	–11.64 ± 4.15	–12.23 ± 3.67	–10.55 ± 4.10*	–12.39 ± 3.56	–10.89 ± 3.98*	–12.48 ± 3.56	–10.23 ± 4.13*	–12.71 ± 4.15	–9.83 ± 3.66*
LASct-c (%)	–17.32 ± 6.55	–18.62 ± 5.04	–15.92 ± 4.72*	–18.74 ± 5.29	–15.89 ± 4.38*	–18.72 ± 5.19	–16.09 ± 4.64	–19.31 ± 5.67	–14.66 ± 3.79*

^#^*p* < 0.0001, * *p* < 0.05, between subgroups of low and high LAVImax, LAVImin, LASr, and LASr-c. Abbreviations: same as Table 1 and Table 2.

**Table 4.** **Univariate and multivariable Cox hazard regression analysis for LA parameters for major adverse cardiovascular events**.

	LAVmax		LAVmin		LASr		LASr-c	
	HR (95% CI)	*p*	HR (95% CI)	*p*	HR (95% CI)	*p*	HR (95% CI)	*p*
Univariate	1.08 (1.00–1.17)	**0.001**	1.27 (1.12–1.38)	< **0.001**	0.79 (0.74–0.84)	< **0.001**	0.89 (0.84–0.94)	< **0.001**
Model 1 adjusted	1.06 (1.02–1.11)	**0.022**	1.25 (1.12–1.37)	< **0.001**	0.82 (0.76–0.89)	< **0.001**	0.99 (0.92–1.05)	0.693
Model 2 adjusted	1.05 (0.97–1.09)	0.340	1.21 (1.08–1.35)	**0.010**	0.81 (0.74–0.89)	< **0.001**	0.98 (0.93–1.05)	0.703
As dichotomous variables	>32.2 vs ≤32.2 mL/m^2^		>16.9 vs ≤16.9 mL/m^2^		<18.50% vs ≥18.50%		<27.00% vs ≥27.00%	
Univariate	2.47 (1.31–4.67)	**0.005**	3.58 (1.65–7.76)	**0.001**	5.23 (2.67–10.25)	< **0.001**	2.27 (1.22–4.23)	**0.010**
Model 1 adjusted	2.14 (1.12–4.12)	**0.022**	3.12 (1.41–6.89)	**0.003**	4.65 (1.85–9.10)	< **0.001**	2.54 (1.34–4.82)	**0.004**
Model 2 adjusted	1.65 (0.84–3.26)	0.147	2.25 (1.03–6.39)	**0.005**	3.95 (1.76–8.86)	< **0.001**	1.48 (0.71–3.10)	0.297

Model 1: preliminarily adjusted model, adjusted for age, SBP, Smoking, HbA1c, triglyceride, CR and GFR. Model 2: fully adjusted model, adjusted for LVEDd and GLS on top of model 1. HR, hazard ratio. The data highlighted in boldface were found to be statistically significant.

Table 4 shows that both LAVImin and LASr were independently associated with MACEs after adjusting for a range of clinical and echocardiographic predictors. A standard deviation increase in LAVImin was linked to an approximately 20% to 30% increase in the risk of reaching the primary endpoint (HR, 1.21; 95% CI, 1.08–1.35; *p* = 0.010). Conversely, a 10 percent increase in LASr was associated with a decreased risk, ranging from approximately 20% to 30%, of failing to achieve this endpoint (HR, 0.81; 95% CI, 0.74–0.89; *p* < 0.001). Furthermore, when analyzed as dichotomous variables, patients exhibiting an elevated LAVImin greater than 16.9 mL/m^2^ demonstrated nearly double the event risk compared with their counterparts without this characteristic (HR, 2.25; 95% CI, 1.03–6.39; *p* = 0.005), whereas those presenting with a reduced LASr below 18.5% faced an even more pronounced escalation in event risk—nearly fourfold higher than individuals with a LASr ≥18.5% (HR, 3.95; 95% CI, 1.76–8.86; *p* < 0.001).

### 3.3 Kaplan-Meier Analysis

**Fig. 2.**
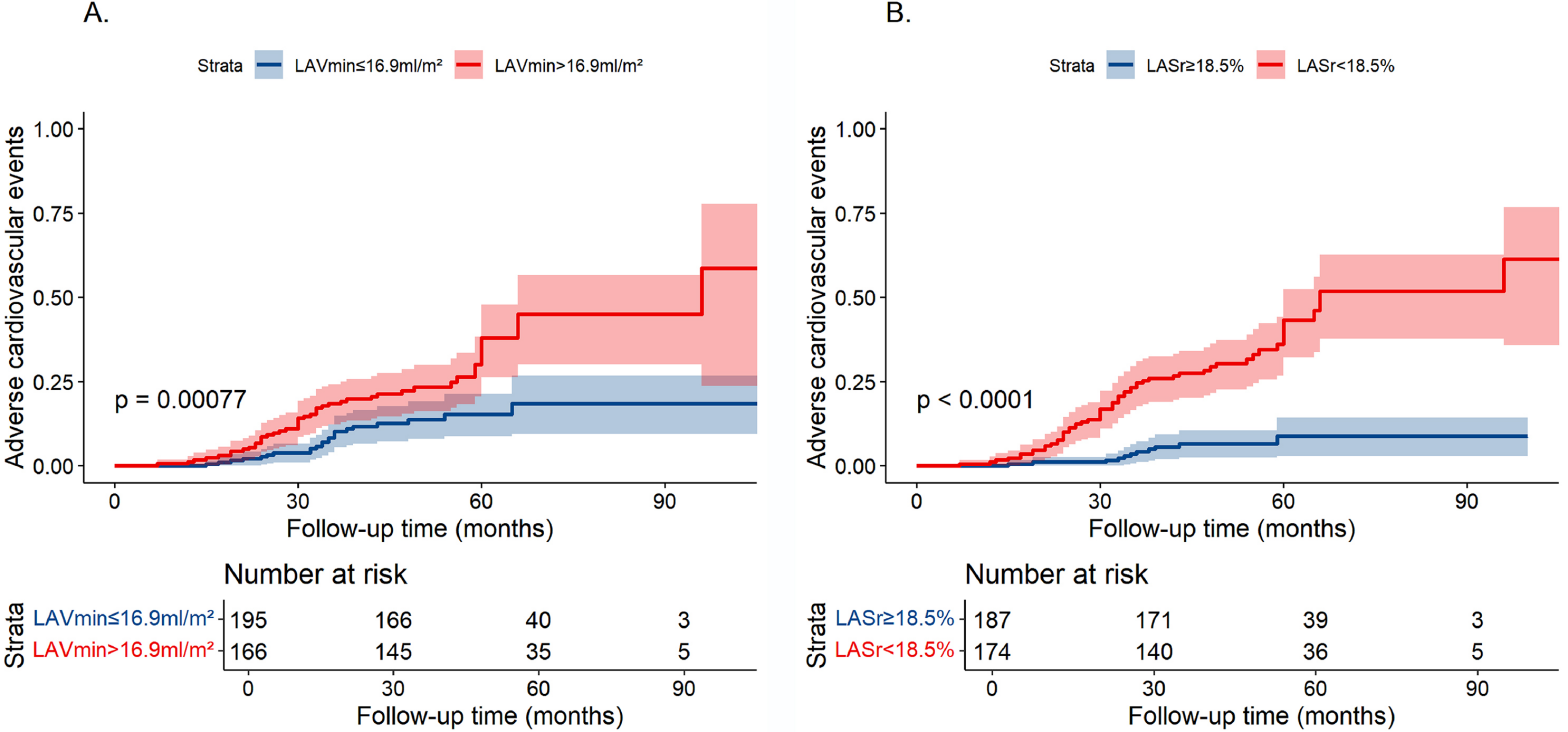
**Illustrates the Kaplan-Meier cumulative incidence rates of primary endpoint events stratified by LAVImin and LASr across the entire cohort**. Patients with enlarged left atrial volumes as denoted by LAVImin exceeding 16.9 mL/m^2^ (A), and diminished LA reservoir function, as indicated by a LASr below 18.5% (B) exhibited a heightened risk for adverse outcomes including coronary heart disease, stroke, congestive heart failure, or cardiac death during follow-up.

Kaplan-Meier analysis revealed that patients with a greater minimum LA volume index (LAVImin >16.9 mL/m^2^) and lower LA strain reserve (LASr <18.5%) faced an increased risk of adverse events during follow-up (*p* < 0.005 for all; refer to Fig. 2).

### 3.4 Subgroup Analysis and Interaction Effects Analysis

**Fig. 3.**
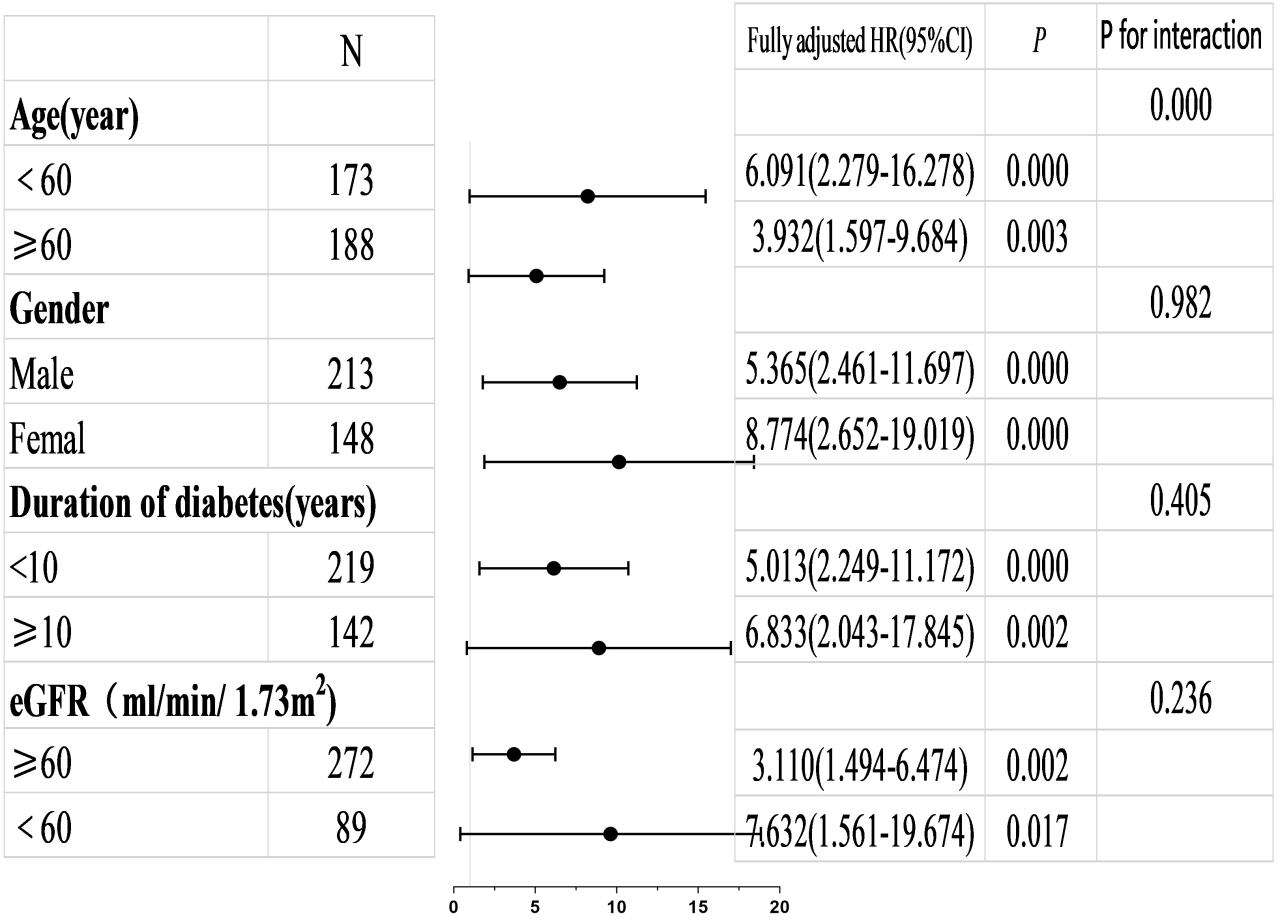
**Illustrate the adjusted HRs for low versus high LASr values (<18.5% vs ≥18.5%) as a prognostic biomarker for MACEs across various patient subgroups**. The HRs presented are derived from a fully adjusted statistical model.

**Fig. 4.**
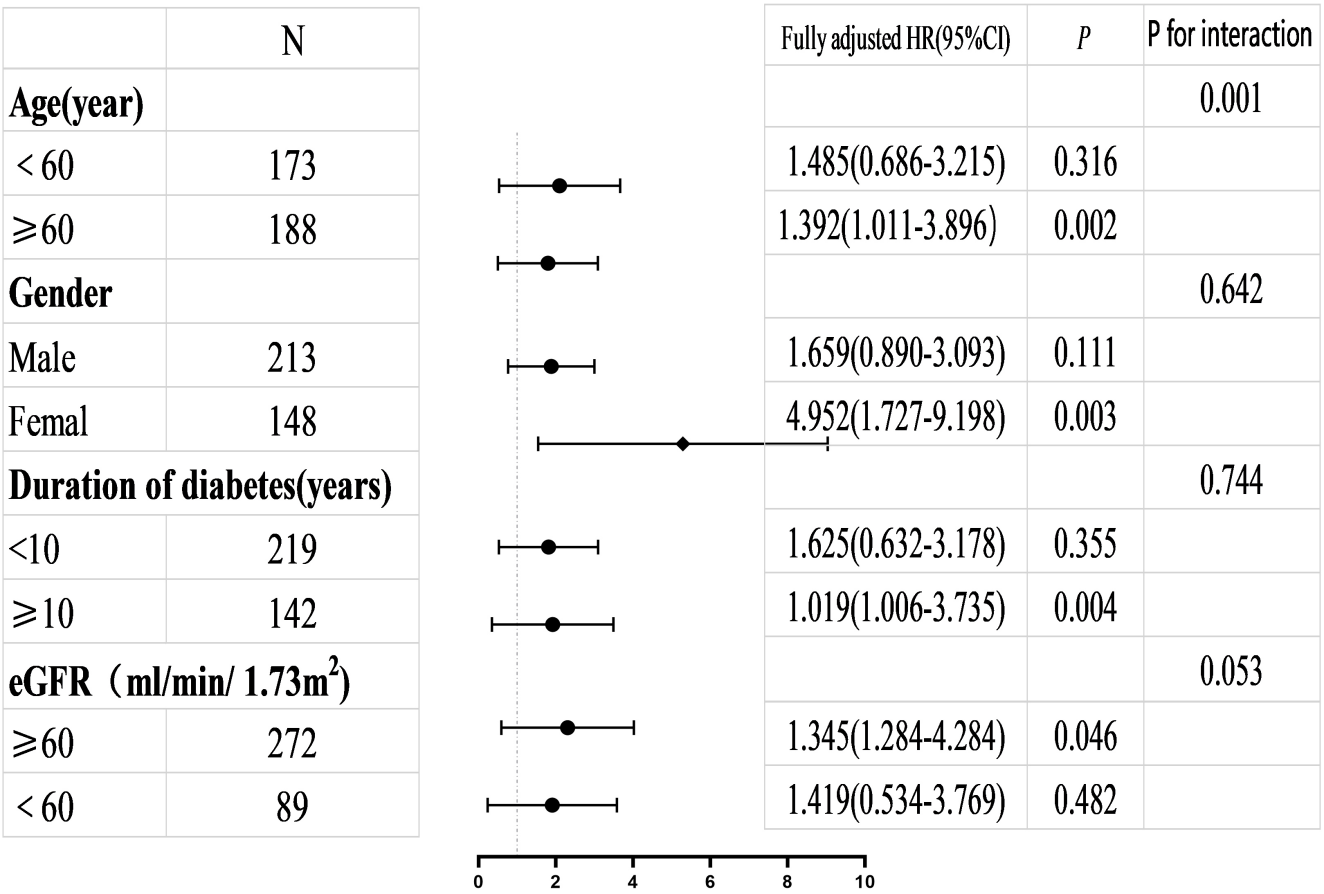
**Illustrate the adjusted HRs high versus low LAVImin values (>16.9 mL/m^2^ vs ≤16.9 mL/m^2^) as a prognostic biomarker for MACEs across various patient subgroups**. The HRs presented are derived from a fully adjusted statistical model.

Subgroup analysis and interaction effects analysis were conducted to further evaluate the associations between MACEs and the parameters LAVImin >16.9 mL/m^2^ and LASr <18.5% across different subgroups. Subgroups were stratified by age, sex, duration of diabetes and GFR. Refer to Figs. 3,4. The risk of MACEs was significantly elevated in patients with LAVImin >16.9 mL/m^2^ within the >60-year-old age group and among females. Patients with LASr <18.5% exhibited a consistently increased risk of MACEs across all subgroups, yielding statistically significant results with consistent trends observed both overall and within subgroups. Interaction analysis revealed that the interaction *p*-value for age was less than 0.05.

### 3.5 The Combined Models of LAVImin and LASr Were Utilized to Predict the Receiver Operating Characteristic (ROC) Curve for MACEs in Patients With DN

**Fig. 5.**
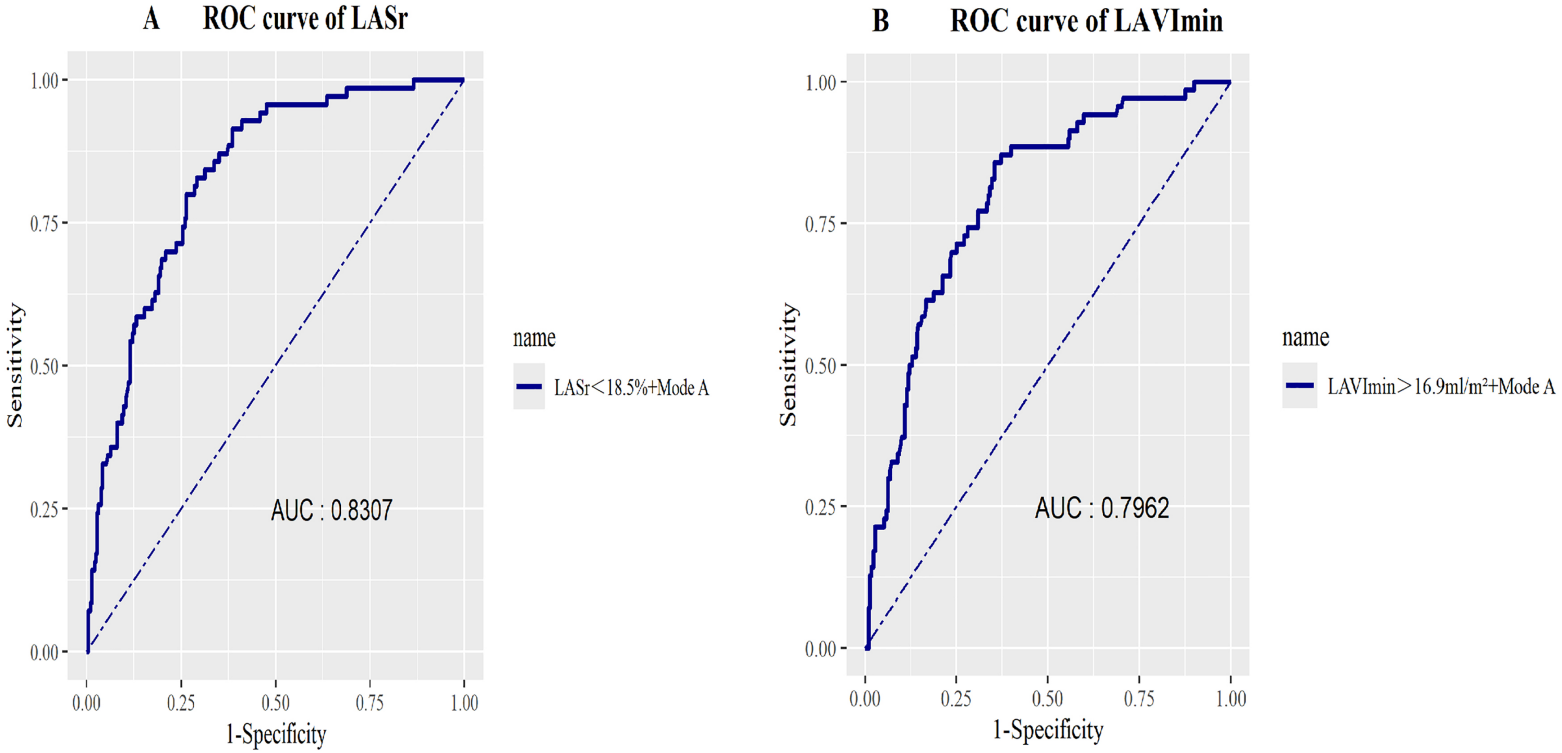
**Illustrates comparison of receiver operating characteristic (ROC) curves and respective area under the curve (AUC) of two nested models for the prediction of MACEs in patients with diabetic nephropathy (DN)**. Model A, includes the basic mode (which included age, SBP, smoking status, HbA1c levels, triglyceride concentrations, CR, GFR, LVEDd, and LVGLS); (A) Incorporate the condition LASr <18.5% into Model A; (B) Incorporate the condition LAVImin >16.9 mL/m^2^ into Model A. LVGLS, left ventricular global longitudinal strain.

By incorporating LASr <18.5% or LAVImin >16.9 mL/m^2^ into the baseline models (which include age, SBP, smoking status, HbA1c level, triglyceride concentration, CR, GFR, LVEDd, and LVGLS), we evaluated model performance through two approaches: (1) comparing the C statistics (area under the curve [AUC]) of the nested models using time-dependent ROC analysis, and (2) conducting a likelihood ratio test within a Cox proportional hazards regression model. The AUC for LASr <18.5% was 0.8307, demonstrating higher specificity and sensitivity compared to LAVImin >16.9 mL/m^2^. Refer to Fig. 5.

Sensitivity analysis showed that when the predicted value increases or decreases by 20%, the values of AUC, sensitivity and specificity all decreased. See **Supplementary Table 3**.

## 4. Discussion

This study demonstrated that LA volume and LA strain were associated with the composite outcomes of cardiac hospitalization, stroke and all-cause death at long-term follow-up in asymptomatic T2DM patients with early CKD. With the occurrence of MACEs, there was an increasing trend in the LA volume indices (LAVImax and LAVImin), whereas the absolute value of the LA strain indices decreased. The structural and functional changes in the LA occur earlier than the changes in the diastolic and systolic functions of the LV. Among the LA parameters tested, LASr consistently showed the strongest associations with outcomes.

DN initiates with glomerular hypertension and hyperfiltration, followed by the emergence of MA, hypertension, overt proteinuria, nephrotic syndrome, and progressive GFR decline, which can lead to significant structural and functional abnormalities in the heart [24]. Research from northern India suggests a progressive decrease in GLS with increasing levels of proteinuria [25]. A previous epidemiological study confirmed that: albuminuria, even at low levels, is associated with adverse cardiac mechanics [26]. Structural, functional, and mechanical alterations in the left atrium serve as sensitive and reliable indicators of cardiac remodeling and function [27]. Our investigation of early DN revealed that with the occurrence of MACEs, there were statistically significant differences in LA volume (LAVImin, LAVImax) and LA strain (LASr, LAScd, LASr-c, LAScd-c) (*p* < 0.05). However, there were no significant differences in the LV size, systolic function parameters (LVEF and GLS), or diastolic function parameters (E/A, E/e’). These findings suggest that the structural and functional changes in the LA occur earlier than the changes in diastolic and systolic functions of the LV.

LA size is closely associated with AF, HF, stroke, and overall mortality postmyocardial infarction [28], making it a robust predictor of cardiovascular events and a crucial biomarker of various CVDs [29]. Animal experiments suggest that LA remodeling in diabetic patients is characterized by enlargement and interstitial fibrosis [30]. Owing to the direct exposure of the LA to LV pressure during end-diastole when the mitral valve is open, the relationship between LAVImin and LVDD is more direct. Study has confirmed that LAVImin increases in early LVDD, whereas LAVImax increases in later stages of LVDD, with the correlation of LAVImin and LVDD being greater than that of LAVImax [31]. Wen *et al*. [32] demonstrated that LAVImin is the LA parameter most strongly correlated with composite and secondary endpoints in AF ablation patients. This study revealed that LAVImin was strongly associated with the cumulative incidence of MACEs in patients with early DN after adjusting for various clinical and echocardiographic predictors (*p* < 0.001). In contrast, there was no significant association between LAVImax and the cumulative incidence of MACEs (*p* = 0.34).

The LA muscle is characterized by its thinness and composition of superficial and deep muscle layers. The superficial muscle, which is transverse in orientation, encircles both the left and right atria. Moreover, the deep muscle consists of longitudinal and circular myocardium, with some ring fibers surrounding the auricle and pulmonary vein orifice to prevent blood reflux during atrial contraction. The intricate arrangement of LA muscle fibers determines their complex movement pattern, resulting in longitudinal and annular strains. 4D Auto LAQ can comprehensively display the longitudinal strain and the circumferential strain of the LA in real time with high sensitivity, reproducibility, and accuracy and reflect the myocardial motion status in detail [33]. This technique is more sensitive to LA dysfunction. accurately, real-time and synchronously estimated the longitudinal and circumferential strain of the LA at each stage.

Myocardial stiffness resulting from hyperglycemia and hyperfiltration is already present in early DN and progresses, leading to imperceptible subclinical diastolic dysfunction and LA fibrosis. Patients with stiff LA can develop diabetic cardiomyopathy (DCM), HF. The study revealed altered gene expression in the central atrial tissue of DN models, with the collagen I and III genes, which are associated with fibrosis, being significantly upregulated. This change not only increased the quantity of collagen fibers in the LA but also altered their composition, leading to myocardial fibrosis and a gradual decline in atrial elasticity and systolic function [34]. The research results suggest that LASr is correlated with atrial *COL1A1* gene expression, which is associated with fibrosis-related gene expression [35]. LA strain is associated with LA myocardial fibrosis [19], patients with low LASr exhibit increased atrial fibrosis-related gene expression, DCM is characterized by myocardial fibrosis and cardiomyocyte remodeling, which substantially increase LA stiffness. This condition not only affects cardiac structure and function but also may accelerate the progression and exacerbation of heart disease [36]. Edwards *et al*. [37] reported that patients with CKD without a history of cardiovascular disease have abnormal strain parameters. Previous studies on CKD and cardiac mechanics have shown that CKD is associated with impaired left atrial reservoir function [38], and that arterial stiffness is related to changes in LA reservoir function (passive filling) [39]. LA reservoir function depends on both LA pressure and compliance but also on LV systolic function [30]. Abnormal LASr is significantly correlated with a poorer functional class defined by the New York Heart Association, even with a normal LAVI [40]. Research indicates that LA strain is negatively correlated with the degree of delay in myocardial enhancement on magnetic resonance imaging [41]. The LASr and LAScd indices exhibit the highest discriminative value in predicting worsening diastolic function [42]. LASr is moderately to strongly correlated with LV end diastolic pressure, and is a reliable marker of LV diastolic function [43, 44]. Our study also revealed that the absolute values of indicators reflecting the LA reservoir and catheter function (LASr, LASr-c, LAScd, and LAScd-c) were lower in patients with DN with MACEs than in those without MACEs (*p* < 0.05). Following initial and comprehensive adjustment for potential mediators, LASr was strongly associated with the cumulative incidence of cardiovascular events in patients with early DN, and consistently exhibited the strongest association with adverse cardiovascular event outcomes (*p* < 0.001). The bivariate analysis indicated that when the LASr was less than 18.5%, the risk of experiencing a MACEs was nearly fourfold greater after adjusting for various clinical and echocardiographic predictors. LASr has incremental value to LAVImin.

For patients with LASr <18.5%, both the full dataset analysis and subgroup analyses yielded statistically significant results, with consistent trends observed across subgroups: the findings were statistically significant for the entire target population, and the results of each subgroup analysis aligned with those of the overall population.

Considering multiple testing, a *p*-value of less than 0.05 for the interaction of age may not be statistically significant. According to recent study, patients diagnosed with early-onset T2DM (age of onset under 40 years) exhibit a markedly greater risk of all-cause mortality than those with late-onset T2DM (age of onset at or above 60 years) [45]. Other study has shown that older age is associated with an increased risk of macrovascular complications and death [46]. However, further investigation is needed to fully understand this interaction.

### Strengths and Limitations

The strengths of this study include the standardized recruitment of high-risk patients, a comprehensive echocardiographic assessment, and the incorporation of 4D Auto LAQ for assessing cardiac mechanics. Structural changes in the left atrium (LAVImin) and dysfunction during the reservoir phase (LASr) are independently correlated with MACEs during long-term follow-up in asymptomatic patients with T2DM with early CKD. Furthermore, the inclusion of LASr significantly improved the predictive value for MACEs in patients with DN. Early identification of structural and strain alterations in cardiac function can provide robust support for comprehensive management strategies and individualized treatment plans for patients with DN.

However, there are a few limitations to this study. The primary limitations of this study include its single-center design, which necessitated multicenter validation with a larger sample size to obtain high-level clinical evidence. Second, recruitment occurred exclusively among patients with a prior history of CKD hospitalization, and the study participants probably represent a higher risk population than those who would be seen exclusively in the outpatient setting. Additionally, this study allowed for the assessment of associations but not causation. Addressing this issue requires more prospective research.

## 5. Conclusions

This study revealed that an increase in LAVImin and a reduction in LASr are independently associated with MACEs in asymptomatic patients with T2DM with early CKD. LASr is the strongest LA parameter associated with prognosis, offering a novel reference index for assessing cardiac function in patients with DN.

## Availability of Data and Materials

The datasets used and/or analyzed during the current study are available from the corresponding author on reasonable request.

## Supplementary Material

Supplementary material associated with this article can be found, in the online version, at https://doi.org/10.31083/RCM27247.
