# Supplementary material for: Association of Left Atrium Remodeling With Major Adverse Cardiovascular Events in Asymptomatic Type 2 Diabetes Patients With Early Chronic Kidney Disease
Source: Rev Cardiovasc Med. 2025 May 21;26(5):27247. doi: 10.31083/RCM27247 (PMC12135644; doi:10.31083/RCM27247)
Supplement: Supplementary file 1 [file 2153-8174-26-5-27247-s1.zip › Supplementary Table 1.docx]

Supplementary Table 1 The ROC analysis evaluated the LA factor of the subject as a single predictor for the MACE threshold

|  | AUC | 95% CI | P value | Cut-off | Sensitivity% | Specificity% |
| --- | --- | --- | --- | --- | --- | --- |
| LAVImin | **0.633** | 0.546 to 0.720 | **0.007** | 16.9ml/m² | 80.50% | 52.60% |
| LAVImax | **0.513** | 0.394 to 0.576 | 0.068 | 32.2ml/m² | 51.22% | 69.53% |
| LAVIpre | 0.484 | 0.388 to 0.579 | 0.740 |  |  |  |
| LA EF | **0.597** | 0.488 to 0.705 | 0.650 |  |  |  |
| LASr | **0.774** | 0.681 to 0.866 | **0.000** | 18.50% | 70.73% | 74.42% |
| LAScd | 0.292 | 0.200 to 0.383 | **0.000** |  |  |  |
| LASct | 0.302 | 0.227 to 0.376 | **0.000** |  |  |  |
| LASr-c | **0.713** | 0.623 to 0.803 | **0.000** | 27.00% | 56.10% | 75.81% |
| LAScd-c | 0.346 | 0.253 to 0.439 | **0.002** |  |  |  |
| LASct-c | 0.407 | 0.309 to 0.504 | 0.058 |  |  |  |

Abbreviations: ROC, Receiver Operating Characteristic Curve; AUC, Area Under Curve; CI: confidence interval.

The rest are the same as in Table 2

The data highlighted in boldface were found to be statistically significant.
