# Supplementary material for: Association of Left Atrium Remodeling With Major Adverse Cardiovascular Events in Asymptomatic Type 2 Diabetes Patients With Early Chronic Kidney Disease
Source: Rev Cardiovasc Med. 2025 May 21;26(5):27247. doi: 10.31083/RCM27247 (PMC12135644; doi:10.31083/RCM27247)
Supplement: Supplementary file 1 [file 2153-8174-26-5-27247-s1.zip › Supplementary Table 2.docx]

Supplementary Table 2 Factors associated with major adverse cardiovascular events at univariate Cox regression analysis (total N = 361)

|  | Major adverse cardiovascular events (70 patients) | | |
| --- | --- | --- | --- |
|  | HR | 95.0% CI | P |
| Age | 1.05 | 1.01-1.13 | **0.036** |
| Sex | 1.95 | 0.88-4.29 | 0.097 |
| BMI | 0.63 | 0.38-1.11 | 0.112 |
| SBP | 1.04 | 1.02-1.06 | **0.001** |
| DBP | 1.00 | 0.92-1.07 | 0.867 |
| Current smokers | 1.05 | 1.01-1.14 | **0.045** |
| Family history of diabetes | 0.93 | 0.85-1.16 | 0.527 |
| Duration of diabetes | 1.00 | 0.96-1.05 | 0.978 |
| UACR | 1.00 | 0.98-1.03 | 0.707 |
| CR | 1.15 | 1.03-1.35 | **0.010** |
| eGFR | 0.91 | 0.79-0.98 | **<0.001** |
| FBG | 1.08 | 0.88-1.33 | 0.434 |
| PBG | 0.97 | 0.83-1.16 | 0.667 |
| HbA1c | 0.82 | 0.63-1.03 | 0.187 |
| BNP | 1.00 | 0.99-1.22 | 0.271 |
| Total cholesterol | 1.08 | 0.84-1.47 | 0.608 |
| Triglyceride | 1.22 | 1.02-1.98 | **0.020** |
| HDL | 0.84 | 0.69-1.02 | 0.078 |
| LDL | 0.97 | 0.63-1.50 | 0.904 |
| E/A | 1.06 | 0.20-5.61 | 0.944 |
| E/e' | 1.03 | 0.91-1.16 | 0.671 |
| LVDd | 1.07 | 1.01-1.17 | **0.023** |
| LVSd | 0.90 | 0.76-1.06 | 0.146 |
| LVEF | 1.04 | 0.94-1.14 | 0.469 |
| LVGLS | 1.15 | 1.01-1.32 | **0.046** |

Abbreviations: Table1, Table 2

The data highlighted in boldface were found to be statistically significant.
