# Supplementary material for: Association of Left Atrium Remodeling With Major Adverse Cardiovascular Events in Asymptomatic Type 2 Diabetes Patients With Early Chronic Kidney Disease
Source: Rev Cardiovasc Med. 2025 May 21;26(5):27247. doi: 10.31083/RCM27247 (PMC12135644; doi:10.31083/RCM27247)
Supplement: Supplementary file 1 [file 2153-8174-26-5-27247-s1.zip › Supplementary Table 3.docx]

Supplementary Table 3: Area Under the Receiver Operating Characteristic Curve for the Predictive Model with Various Cutoff Values

|  | AUC | Sensitivity% | Specificity% |
| --- | --- | --- | --- |
| LASr |  |  |  |
| <18.5% vs ≥18.5% | 0.831 | 70.83% | 85.95% |
| <22.1% vs ≥22.1% | 0.798 | 61.45% | 87.14% |
| <14.8% vs ≥14.8% | 0.809 | 86.11% | 64.28% |
| LAVImin |  |  |  |
| ＞16.9 vs≤ 16.9 ml/m² | 0.796 | 64.58% | 86.14% |
| ＞20.2 vs≤ 20.2 ml/m² | 0.793 | 68.40% | 80.01% |
| ＞13.5 vs≤ 13.5 ml/m² | 0.791 | 71.18% | 75.17% |

Abbreviations: AUC, Area Under Curve; The rest are the same as in Table 2
